# Cornual Epithelioid Trophoblastic Tumor: A Report of a Rare Case and Literature Review

**DOI:** 10.7759/cureus.81340

**Published:** 2025-03-28

**Authors:** Nawal A Alharbi, Ghada I Abdulhamid, Salafa Albaghli, Ayman M Husain

**Affiliations:** 1 Obstetrics and Gynecology, King Saud Medical City, Riyadh, SAU; 2 Pathology, King Saud Medical City, Riyadh, SAU

**Keywords:** beta-human chorionic gonadotropin (β-hcg), chemotherapy, cornual ectopic pregnancy, epithelioid trophoblastic tumor (ett), gestational trophoblastic neoplasm, surgery

## Abstract

Cornual ectopic pregnancy is a rare type of ectopic pregnancy that occurs in the cornua of the uterus. This condition poses significant risks, including rupture and severe hemorrhage, making early diagnosis and management crucial.

Gestational trophoblastic neoplasm (GTNS) is a spectrum of malignant diseases including invasive mole, choriocarcinoma, placental site trophoblastic tumor (PSTT), and epithelioid trophoblastic tumor (ETT).

ETT is a rare type of gestational trophoblastic tumor. While ETT shares some similarities with other trophoblastic tumors, it has distinct histological features, clinical behaviors, associations, and treatment approaches that set it apart.

We present a rare case involving a 38-year-old Saudi woman diagnosed with a cornual ectopic pregnancy, which was later histopathologically confirmed as ETT. Initial treatment included systemic and local methotrexate, but the patient exhibited persistently elevated beta-human chorionic gonadotropin (β-hCG) levels, necessitating surgical intervention. A wedge resection of the cornual ectopic mass was performed, and histopathological analysis confirmed the diagnosis of ETT. Postoperative imaging indicated small bilateral pulmonary nodules that raised concerns for metastasis. The patient underwent multi-agent chemotherapy, beginning with methotrexate and later transitioning to dactinomycin due to insufficient decline in β-hCG levels. After three cycles of dactinomycin, β-hCG levels returned to normal.

This case emphasizes the diagnostic difficulties associated with ETT and highlights the critical role of histopathological examination for patients with persistent β-hCG elevation. In this instance, surgical resection followed by multi-agent chemotherapy proved to be an effective treatment approach.

We reported this case because of its rarity and diagnostic and therapeutic challenges.

## Introduction

Cornual ectopic pregnancy is a rare and dangerous form of ectopic pregnancy, representing 2-4% of all ectopic cases [1]. It occurs at the uterine cornu, where the fallopian tube connects to the uterus. This type carries a high risk of rupture and severe hemorrhage due to the area’s abundant blood supply and thin myometrial wall.

Gestational trophoblastic neoplasms (GTNs) include a variety of malignant trophoblastic disorders such as invasive mole, choriocarcinoma, placental site trophoblastic tumor (PSTT), and epithelioid trophoblastic tumor (ETT). ETT, a rare subtype, constitutes only 1-2% of all GTNs [2,3]. Initially referred to as “atypical choriocarcinoma,” ETT was officially classified by the World Health Organization (WHO) in 2003 [4,5]. Unlike choriocarcinoma, which arises from syncytiotrophoblasts and cytotrophoblasts, ETT originates from chorionic-type intermediate trophoblasts and displays distinct histopathological characteristics [5,6].

Typically, ETT occurs in the lower uterine segment or cervix, but it is infrequently found in the uterine cornua. Diagnosis remains challenging due to overlapping clinical features with other GTNs and ectopic pregnancies. This case report discusses an unusual manifestation of ETT that mimicked a cornual ectopic pregnancy, detailing its diagnosis and management.

## Case presentation

A 38-year-old multiparous Saudi woman (G4P3) presented to the emergency department three months after childbirth, complaining of abdominal pain and irregular vaginal bleeding. Her obstetric history included two vaginal deliveries and a preterm cesarean section at 32 weeks due to placenta previa accompanied by antepartum hemorrhage.

**Figure 1:**
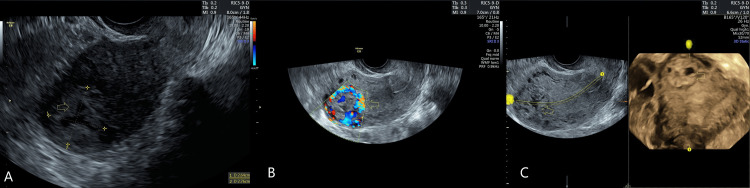
Ultrasound images shows the cornual ectopic. (A) shows the cornual ectopic. (B) shows the increased vascularity of the mass. (C) shows the three-dimensional ultrasound of the mass.

Upon examination, the patient was hemodynamically stable. Laboratory results indicated a hemoglobin level of 12 g/dL and a significantly elevated serum beta-human chorionic gonadotropin (β-hCG) level of 24,000 mIU/mL. A transvaginal ultrasound revealed a 30 × 27 mm right cornual mass with peripheral vascularity, indicative of a gestational sac without an embryo. A diagnosis of cornual ectopic pregnancy was established (Figure 1).

Initial management

The patient received three doses of intramuscular methotrexate; however, her β-hCG levels showed only a suboptimal decline from 24,000 to 10,864 mIU/mL. A fourth dose of locally injected methotrexate into the suspected gestational sac led to a temporary drop in β-hCG to 3,759 mIU/mL, but levels subsequently rose again, reaching 6,708.83 mIU/mL, prompting the need for surgical intervention.

Surgical findings and histopathology

**Figure 2:**
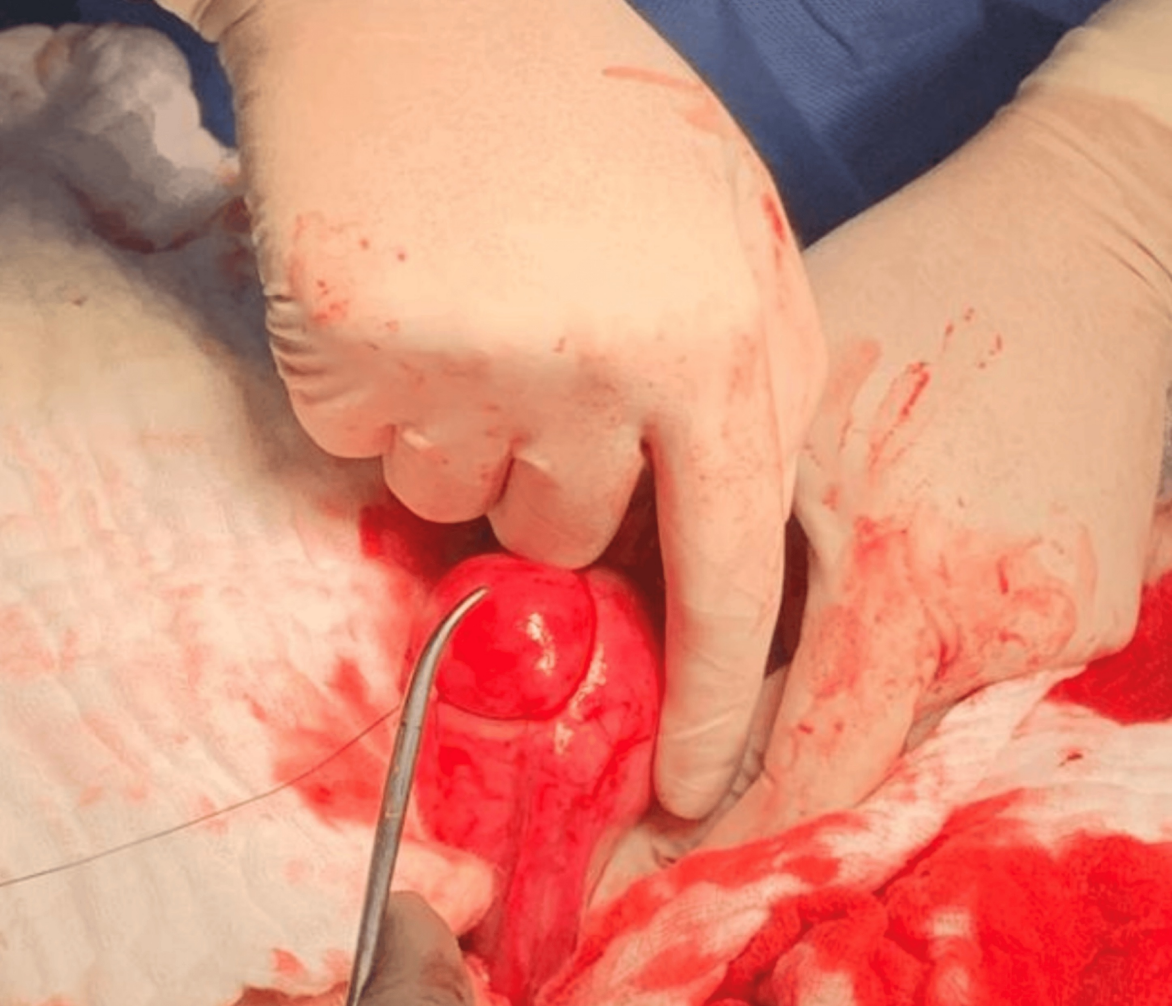
Intraoperative finding (cornual ectopic pregnancy). Cornual ectopic pregnancy was managed with wedge resection.

The patient underwent laparotomy with wedge resection of the cornual mass, followed by primary repair of the uterus (Figure 2).

**Figure 3:**
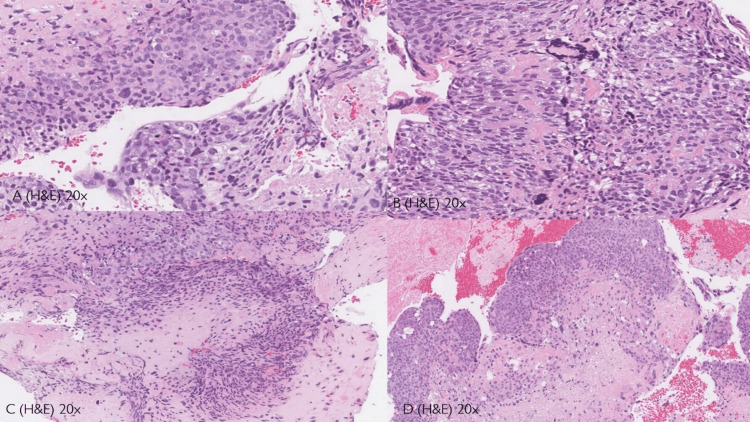
Histopathology analysis (A) Tumor cells have a moderate amount of finely granular, eosinophilic to clear cytoplasm with distinct cell membranes and round nuclei with small nucleoli. (B) Moderate nuclear atypia is seen in most of the tumors with occasional syncytiotrophoblastic. (C) Eosinophilic hyaline material is characteristically present in the center of some tumor nests. (D) Extensive or geographic necrosis

Histopathological examination revealed monomorphic cells arranged in a nested pattern, eosinophilic cytoplasm, and extensive necrosis, consistent with ETT (Figure 3).

**Figure 4:**
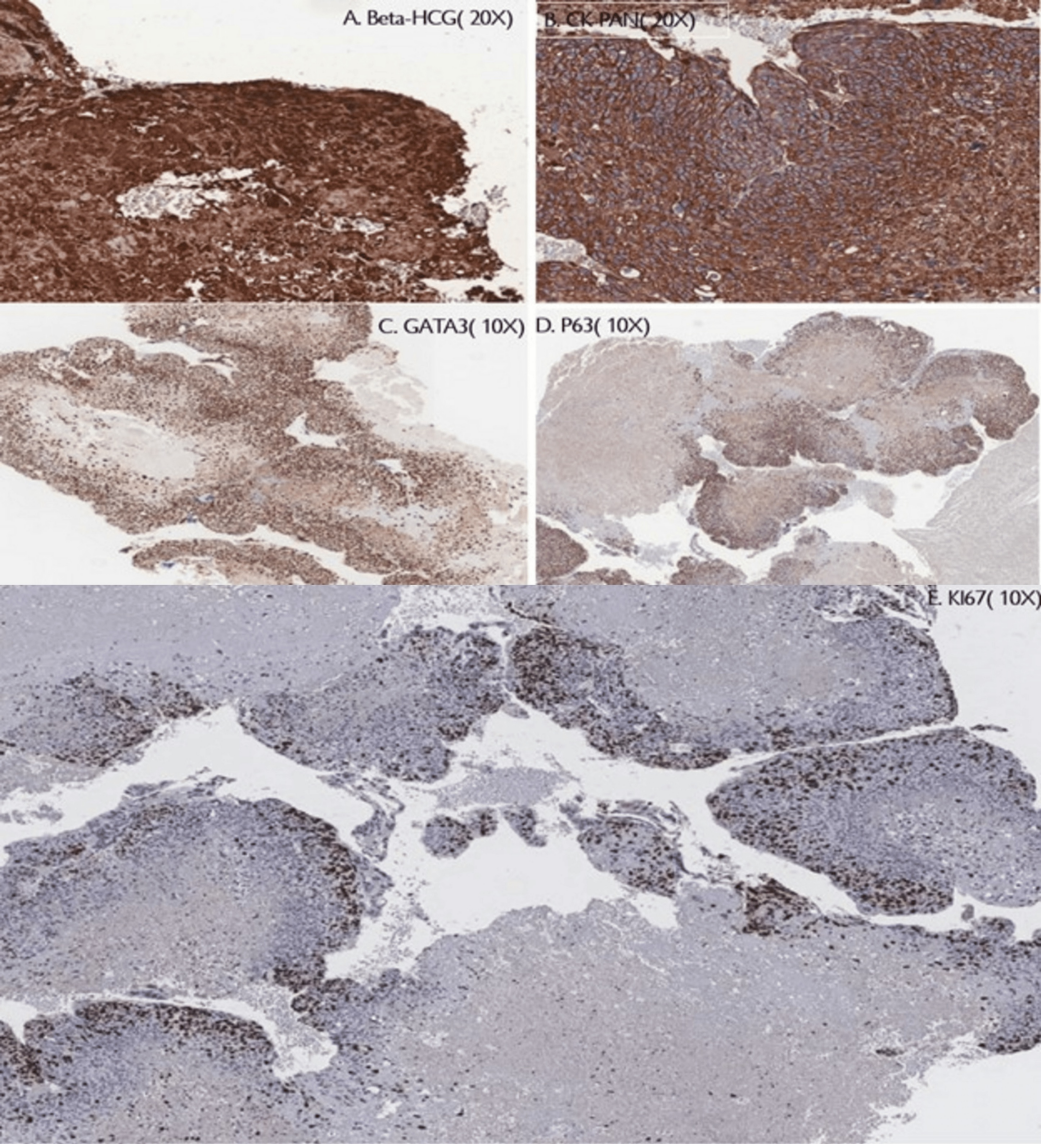
Immunohistochemical stain. The tumor cells are positive for β-hCG (A), CK-Pan (B), GATA3 (C), p63 (D) and negative for hPL, INHIBIN, SALL4, and PAX8. KI67 labeling index 10% (E). hPL: human placental lactogen; β-hCG: beta-human chorionic gonadotropin; CK-Pan: cytokeratin

Immunohistochemical staining was positive for β-hCG, CK-Pan, GATA3, and p63, while negative for hPL, inhibin, SALL4, and PAX8 (Figure 4).

Postoperative course and chemotherapy

**Figure 5:**
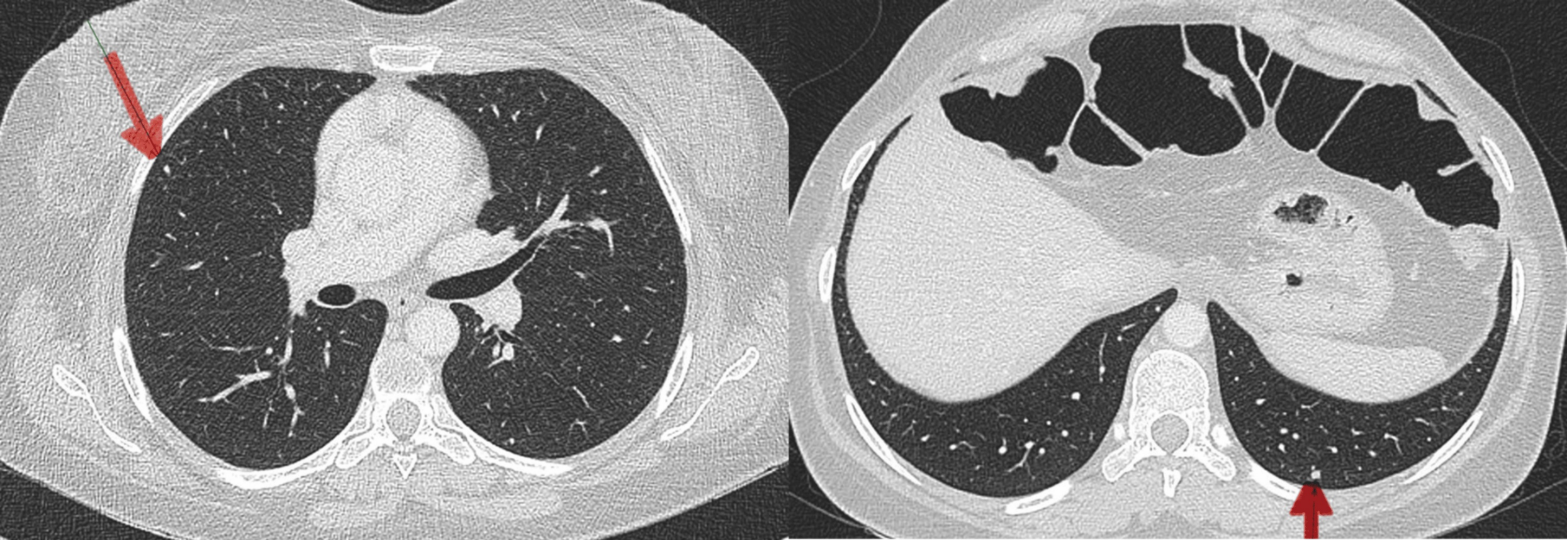
CT scan images. CT scan images showing bilateral small pulmonary nodules. The red arrows indicate small bilateral pulmonary metastases.

A CT scan of the abdomen and pelvis was normal; however, chest imaging detected bilateral small pulmonary nodules, suggesting metastasis (Figure 5).

The patient was referred to gynecologic oncology and initially underwent five cycles of methotrexate. Due to plateauing β-hCG levels, a switch to dactinomycin was made. Following three cycles of dactinomycin, her β-hCG levels decreased to below 2.5 mIU/mL, and she continues to be monitored by the oncologist.

## Discussion

Cornual pregnancies are associated with a high risk of rupture and hemorrhage, with a mortality rate of 2-2.5% [7]. Management approaches may vary based on clinical scenarios, including medical therapy, cornual resection, or hysterectomy [8].

The incidence of GTNs in Asian women is much higher compared to non-Asian women [9]. Our patient was aged 38 years, an age consistent with that of reports that ETT occurs primarily in women of reproductive age with a median age of 32 years. Term or preterm delivery was the most common type of antecedent pregnancy [10]. The majority of cases (67%) occur after full-term pregnancy [11]. This patient's antecedent pregnancy was preterm, and delivery was at 32 weeks gestation by cesarean section because of vaginal bleeding, which was due to placenta previa.

Diagnosis of ETT often faces delays because of its rarity and nonspecific clinical presentation, which can resemble ectopic pregnancy or choriocarcinoma. Histologically, ETT is distinct from choriocarcinoma, characterized by monomorphic intermediate trophoblastic cells, geographic necrosis, and a hyaline-like extracellular matrix; confirmation of ETT requires immunohistochemistry [12-14].

Surgical excision is the preferred treatment for localized ETT, as chemotherapy alone is frequently ineffective. For metastatic cases or those with persistently elevated β-hCG levels, multi-agent chemotherapy is advised. Our patient experienced a suboptimal response to methotrexate monotherapy but showed a positive response to dactinomycin, resulting in normalized β-hCG levels.

ETT has a higher mortality rate (10-24%) compared to post-molar GTN (6.5%) and choriocarcinoma (13.4%) [4,5,14]. Due to the potential for delayed diagnosis and resistance to monotherapy, early surgical intervention combined with multi-agent chemotherapy is vital for optimal outcomes.

## Conclusions

This case illustrates an exceptionally rare presentation of ETT that was initially misdiagnosed as a cornual ectopic pregnancy. The diagnostic challenges associated with ETT highlight the necessity for histopathological and immunohistochemical verification in patients with persistently elevated β-hCG levels. While surgical resection is the primary treatment method, combined chemotherapy is essential for cases that are metastatic or resistant to initial treatment. Early identification and collaborative management are critical for enhancing patient prognosis.
